# Sensory and Instrumental Characterization of Parmigiano Reggiano Protected Designation of Origin Cheese Obtained from Milk of Cows Fed Fresh Herbage vs. Dry Hay †

**DOI:** 10.3390/foods14101781

**Published:** 2025-05-17

**Authors:** Mara Antonia Gagliano, Matilde Tura, Francesca Soglia, Chiara Cevoli, Sara Barbieri, Giacomo Braschi, Alessandra Bendini, Tullia Gallina Toschi, Massimiliano Petracci, Enrico Valli

**Affiliations:** 1 Department of Agricultural and Food Sciences, Alma Mater Studiorum—Università di Bologna, Viale Fanin 40, 40127 Bologna and Piazza Goidanich 60, 47521 Cesena, Italy; maraantonia.gagliano@unibo.it (M.A.G.); francesca.soglia2@unibo.it (F.S.); chiara.cevoli3@unibo.it (C.C.); sara.barbieri@unibo.it (S.B.); giacomo.braschi2@unibo.it (G.B.); alessandra.bendini@unibo.it (A.B.); tullia.gallinatoschi@unibo.it (T.G.T.); m.petracci@unibo.it (M.P.); enrico.valli4@unibo.it (E.V.); 2 Interdepartmental Centre for Industrial Agrofood Research, Alma Mater Studiorum—Università di Bologna, via Bucci 336, 47521 Cesena, Italy

**Keywords:** cheese, protected designation of origin, diet, sensory analysis, quantitative descriptive analysis, color, volatile fraction

## Abstract

Using a multi-analytical approach, this investigation characterized Parmigiano Reggiano PDO cheese produced with milk from dairy cows fed different diets. Ten samples of Parmigiano Reggiano PDO cheese, aged for 24 months, were produced with milk from dairy cows fed only dry hay (P-DH; N = 6) or a diet with part of the dry hay replaced with fresh herbage (P-FF; N = 4). Instrumental (Flash GC-FID) analysis of the volatile fraction, image analyses, and sensory quantitative descriptive analysis (QDA^®^) were carried out. The Parmigiano Reggiano cheese belonging to the P-FF group showed a higher intensity of yellow than P-DH for both sensory and image analyses. Regarding the volatile profiles, no differences were observed related to the two experimental groups, while sensory analyses allowed for some discrimination, in particular color and aroma attributes. Instrumental and sensory characterization can be used to obtain a unique analytical profile for Parmigiano Reggiano PDO cheeses produced with milk from dairy cows fed different forage sources and help to define the quality and authenticity of this typical high-value food product.

## 1. Introduction

Products with a Protected Designation of Origin (PDO) are highly valued for their strong connection to specific geographical areas, traditional production methods, and local expertise. Parmigiano Reggiano (PR), one of the most famous PDO cheeses worldwide, is an extra-hard cheese made from raw bovine milk in Northern Italy. Its production adheres to strict regulations established by the Parmigiano Reggiano Cheese Consortium, which also oversees its compliance. This careful process ensures that PR retains its quality, distinct flavor, and global reputation as one of the finest cheeses [1,2,3].

One of the methods used to assess the quality of PR is sensory analysis. This technique involves evaluating the cheese’s organoleptic characteristics, such as appearance, aroma, flavor, and texture, to ensure that it meets the high-quality standards established by the Consortium [4]. The sensory characteristics of PR cheese are influenced by several factors, including the diet of cows. Numerous studies have established that the inclusion of forage in the diets of dairy cows can significantly influence the physicochemical and sensory characteristics of both milk and cheese [5,6]. Pasture-based feeding has been shown to alter milk composition, imparting unique organoleptic properties compared with diets primarily based on silage or cereal concentrates [2,7]. This correlation between forage type and milk composition is largely attributed to the transfer of specific plant-derived compounds into the milk—and ultimately into the cheese—via a carry-over process [5]. For example, cheese manufactured by milk from cows fed dry hay will have a whiter color compared with that produced from cows fed fresh forage [8]. Moreover, forage type and form may significantly influence ruminal responses which in turn impacts the milk fat content and other milk components [9]. In addition, biochemical processes, influenced by environmental conditions, can cause yellowing or browning of cheese paste. Aging also plays a crucial role in the sensory quality of the product [10]. Among the distinctive sensory characteristics, the presence of tyrosine crystals, visible through visual analysis, increases following proteolytic processes. Fresh milk, yogurt, whey, melted butter, and rind are among the most recognized flavors. Occasionally, vegetal and fruity notes can also be perceived, accompanied by hints of meat broth [4]. As aging continues, the cheese becomes drier and crumblier, while the flavor intensifies, developing a spicier note and revealing aromas of spices and dried fruit. Among the many techniques available to perform descriptive sensory evaluation, Quantitative Descriptive Analysis (QDA^®^) stands out as one of the most advanced methods. This approach involves a group of trained evaluators who systematically analyze various sensory properties of a product, such as aroma, taste, texture, and appearance. Using standardized scales, QDA^®^ provides a detailed and structured understanding of sensory characteristics, making it an invaluable tool for food development and quality control. Additionally, sensory wheels are often used as visual aids to summarize sensory attributes, serving as a practical resource for training evaluators and ensuring consistency in assessments [11].

The aroma profile of cheese, including PR, is the result of complex interplay of volatile organic compounds (VOCs). While each of these compounds on its own might not fully capture the essence of the cheese’s scent, together, they combine to create the distinctive profile that defines PR [12]. The interaction of specific chemical compounds results in the complex, nutty, and savory aroma that distinguishes PR, contributing to its status as a highly esteemed and globally recognized cheese. More specifically, aldehydes play a key role in the development of its characteristic flavor, while esters contribute fruity and subtly sweet notes, and ketones enhance the buttery and creamy characteristics [13].

Moreover, one of its defining sensory attributes is color. It typically ranges from a pale straw-yellow to a deeper golden hue, depending on factors such as the milk type, the cows’ diet, and the aging process. This natural coloration is influenced primarily by the carotenoids present in the milk, which are derived from dietary herbage and hay. These pigments give the cheese its warm, creamy tones, reflecting its natural production process [14]. Various studies have been carried out on different types of dairy products focusing on features such as microbiological aspects, texture, and aromatic profiles [15].

In this regard, all these characteristics can be investigated through a multidisciplinary approach, combining sensory analysis, microbiological analysis, and advanced instrumental techniques. Such an approach allows for the development of a comprehensive analytical profile of each cheese and plays a critical role in ensuring quality control and verifying the authenticity of high-value dairy products [16]. In fact, by evaluating key sensory attributes alongside chemical, physical, and biochemical parameters, this approach provides robust tools for the protection, classification, and enhancement of traditional cheese varieties [17].

Moreover, it is very important to adopt such a multidisciplinary approach to comprehensively address the various factors that influence quality in order to provide consumers with a broader confidence in product quality. Furthermore, this allows food industry operators to gain a deeper understanding of the various aspects that influence consumer behavior at the time of purchase [18].

The aim of the present study is to characterize PR PDO cheeses obtained from the milk of dairy cows fed dry hay with those produced from the milk of cows fed dry hay with integration of fresh herbage. To combine the ability of electronic senses with those of the human senses, and to evaluate the safety of cheese, sensory and instrumental analyses were performed.

## 2. Materials and Methods

### 2.1. Ethical Approval

This study was approved by the Alma Mater Studiorum—Università di Bologna Bioethical Committee (prot. no. 0057910, date 3 March 2023).

### 2.2. Samples

Ten batches of Parmigiano Reggiano PDO cheese were analyzed to assess the sensory profile and identify possible differences in the aroma profile and visual quality using instrumental methods. Microbiological parameters were also used to assess sample safety. The cheeses were produced using milk from dairy cows raised indoors fed identical diets, except for forage: only dry hay or dry hay with fresh herbage. The batches were labeled P-DH (Parmigiano Reggiano from cows fed 15–18 kg of dry hay per cow per day) and P-FF (Parmigiano Reggiano from cows fed 40 kg of fresh herbage per cow per day, plus 9–12 kg of dry hay). Cheeses were shipped by the Parmigiano Reggiano PDO Consortium (“Consorzio del Formaggio Parmigiano Reggiano”, Reggio Emilia, Italy) to the University of Bologna (Department of Agricultural and Food Sciences, Cesena, Italy) for sensory, instrumental, and microbiological analyses. The farming and production processes followed a unified standard document. Both experimental groups were aged for 24 months. A limited number of samples were used in the present study due to the difficulty in identifying dairies that have separated the milk obtained from cows fed with a fresh herbage supplement from that produced by cows whose diet consists solely of dry forage. Each sample consisted of a single piece of cheese from the same wheel, produced from a bulk quantity of milk to replicate commercial conditions, totaling 2 kg per batch. Before analysis, samples were stored under vacuum at 4 °C.

### 2.3. Sensory Analysis

#### 2.3.1. Panel Selection

Nine assessors (6 female, age 25–60 years) were recruited from staff, PhD students, and research fellows at the Department of Agricultural and Food Sciences (Alma Mater Studiorum—Università di Bologna). All assessors had previous training in sensory descriptive analysis of different food products. More details can be found in Section 2.3.2 (sensory vocabulary) and in Section 2.3.4 (training procedure).

#### 2.3.2. Sensory Vocabulary

The selection of descriptors was conducted through an open discussion in 4 preliminary sessions, each one lasting 50 min, according to descriptive analysis [19]. During these sessions, each panelist received five samples per discussion and elicited specific sensory attributes according to appearance, aroma, taste and texture. The panel, with the support of the panel leader, determined whether any descriptors were redundant (removing them from the profile sheet) or if additional attributes should be included. A final list of 13 attributes was established, categorized into visual, olfactory, taste, and texture descriptors, as reported in Table 1.

#### 2.3.3. Evaluation Sheet/Procedure

**Figure 1:**
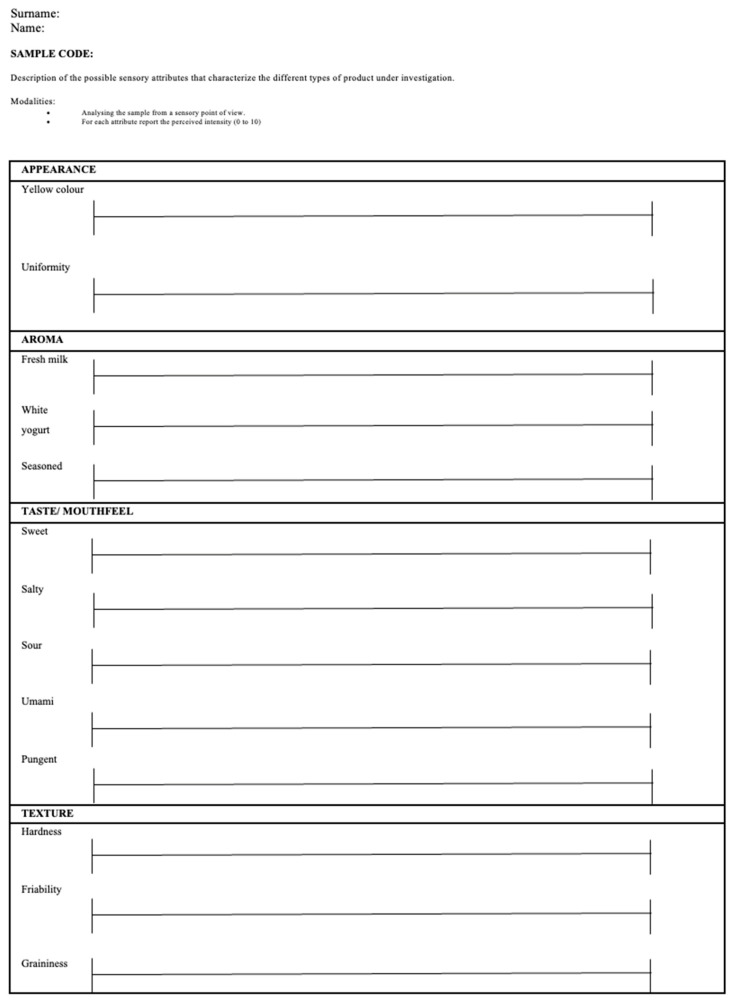
Sensory profile sheet specifically developed for Parmigiano Reggiano PDO cheese. The intensity of each attribute was evaluated with the use of a 100 mm unstructured scale with two anchor points: 0 (not perceivable) and 100 (extremely high).

Thirteen attributes were included in the sensory sheet based on their frequency of elicitation. The evaluation sheet is shown in Figure 1. In order to facilitate and support the consensus of the assessors, reference standards were developed for some of the 13 descriptors, specifically those for which the panelists were not yet aligned in their sensory evaluation (Table 1).

#### 2.3.4. Training Procedure

The assessors participated in 13 training sessions on rating intensities, each lasting approximately one hour. The samples were presented in a randomized order; to limit sensory fatigue, a maximum of 4 samples per session were provided. The evaluation method was performed according to ISO 13299:2010 [20]. Each sample, weighing approximately 4 g (3 pieces per sample), was alphanumerically coded and served in white plastic dishes. The presentation of the samples was randomized. The intensity of each attribute was measured using an unstructured 100 mm scale, ranging from 0 (not perceivable) to 100 (maximum perceivable intensity), with predefined anchor points for each attribute. The samples were tested at room temperature. Assessors were asked to analyze the samples considering the following order: appearance, olfactory, and retronasal characteristics, taste and mouthfeel, and texture. To monitor the panel’s performance, PanelCheck software (ver. 1.4.2; Nofima, Tromso, Norway) was applied.

### 2.4. Image Analysis

The samples underwent image analysis to evaluate differences in color using an instrumental technique. The instrumental measurement of appearance was performed with an “electronic eye” (Visual Analyzer VA400 IRIS, Alpha MOS, Toulouse, France), a high-resolution charge-coupled device (CCD) camera (2592 × 1944 pixels) equipped with a photo sensor capable of capturing 16 million colors. The instrument was equipped with two sets of fluorescent tube lights (2 × 2) with a color temperature of 6700 K; however, only the overhead lighting was used to capture images. Samples were placed on a white plastic tray inside the device’s enclosed light chamber, ensuring uniform light diffusion. The CCD camera then captured an image of each sample. Before image acquisition, the instrument was calibrated using a certified color reference card (ColorChecker Classic, x-Rite, Grand Rapids, MI, USA).

### 2.5. Flash GC-FID Analysis of the Volatile Fraction

The analytical conditions applied were previously detailed by Palagano et al. (2020) [21]. In particular, the analysis of volatile compounds was performed using the Flash GC—FID Electronic Nose Heracles II (Alpha MOS, Toulouse, France), which is based on ultra-fast gas chromatography technology. The vial was placed in a shaker oven for 20 min at 40 °C and agitated at 500 rpm. Subsequently, 5 mL of the headspace was collected and introduced into a splitless injector (injector temperature: 200 °C; injection speed: 100 µL/s; carrier gas flow: 30 mL/min) to ensure rapid transfer of the sample to the trap. The analytes were adsorbed onto a Tenax^®^ TA trap maintained at 40 °C for 60 s. The syringe temperature was set at 70 °C. Desorption was then carried out by increasing the trap temperature to 240 °C over 93 s. The desorbed sample was injected (carrier gas pressure at the column head: 40 kPa) and split (split flow: 5 mL/min) between two columns: a non-polar column (MXT-5; 5% diphenyl, 95% methylpolysiloxane; 10 m length; 180 μm i.d.) and a polar column (MXT-1701; 14% cyanopropylphenyl, 86% dimethylpolysiloxane; 10 m length; 180 μm i.d.). The thermal program began at 40 °C (held for 2 s), increased to 80 °C at a rate of 1 °C/s, and then to 250 °C at 3 °C/s. Hydrogen was used as the carrier gas, with pressure increasing from 40 kPa to 64 kPa at a rate of 0.2 kPa/s. A flame ionization detector (FID) was installed at the end of each column (detector temperature: 260 °C), and the signal was digitized at 0.01 s intervals.

### 2.6. Data Analysis

For sensory results, the intensity data collected from the trained panel were processed using PanelCheck software (version 1.4.2; Nofima, Tromsø, Norway), an open-source tool available for free download at http://www.panelcheck.com (accessed on 22 December 2022). The evaluation of the panel’s performance was proposed by Tomic et al. (2010) [22]. A mixed-model three-way ANOVA was performed to assess the importance of each descriptor in detecting significant sensory differences among samples. Only attributes with a significance level of *p* ≤ 0.05 were considered for further analysis. In order to represent the sensory profile of samples, the average scores for each attribute for each sample were determined and reported as spied plots using Excel (version 16.96, Microsoft 365, Redmond, WA, USA). Principal component analysis (PCA) was applied using medians of sensory attributes to assess potential groupings based on P-FF and P-DH. Furthermore, evaluating the loadings provided insights into the impact of attributes on discrimination (PLS Toolbox for MATLAB R2023a; Eigenvector Research, Inc. USA, MathWorks Inc., Natick, MA, USA). For image analysis, the software provided with the instrument (Alphasoft, version 14.0, Alpha MOS, Toulouse, France) allowed for the color spectra to be grouped in a range of 16 bits for each RGB coordinate, resulting in 4096 variables, reported by the software as histograms. The proportion of each color in the analyzed image, on a fixed scale of 4096 colors, is represented as a percentage and was used to analyze the data from PR samples using principal component analysis (PCA).

Lastly, the 43 main peaks detected in the first Flash GC-FID chromatogram, obtained from the first column (MXT5), were automatically selected and integrated by the Alphasoft software version 14.5. Starting from the full Flash GC-FID chromatograms (Pareto scaling and mean cantering pretreatments) and the 43 main peaks (mean centering pretreatment), PCA was used as an explorative technique to visualize samples according to the type of feeding (PLS Toolbox for MATLAB R2023a; Eigenvector Research, Inc., Manson, WA, USA, MathWorks Inc., Natick, MA, USA).

## 3. Results

### 3.1. Sensory Analysis

**Figure 2:**
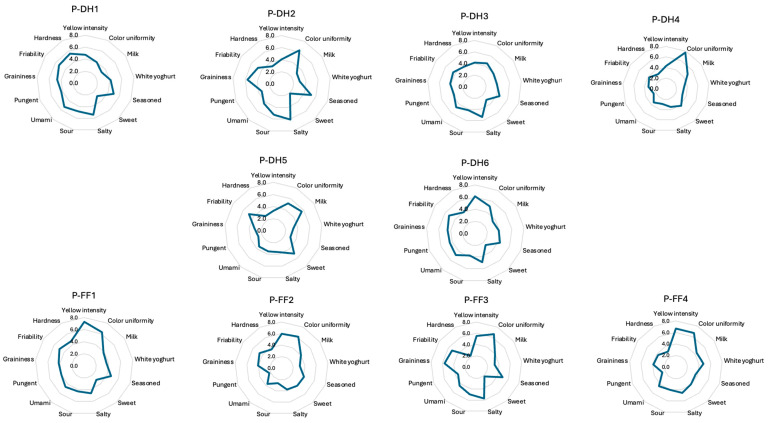
Spider graphs representing the sensory profile of the 10 PR samples (from P-DH1 to P-DH6 and P-FF1 to P-FF4) analyzed. P-DH: PR samples from cows fed with dry hay; P-FF: PR samples from cows fed with a supplementation of fresh herbage.

The results of the analysis with three-way ANOVA determined the alignment (*p* < 0.05), discriminative ability (*p* > 0.05), and repeatability of the panel (*p* < 0.05), for which the panel was considered trained. Figure 2 shows the spider graphs of the sensory profile of the 10 samples. In this regard, samples P-FF1, P-FF2, and P-FF4 had higher color intensities and uniformities compared with those in the P-DH group.

**Figure 3:**
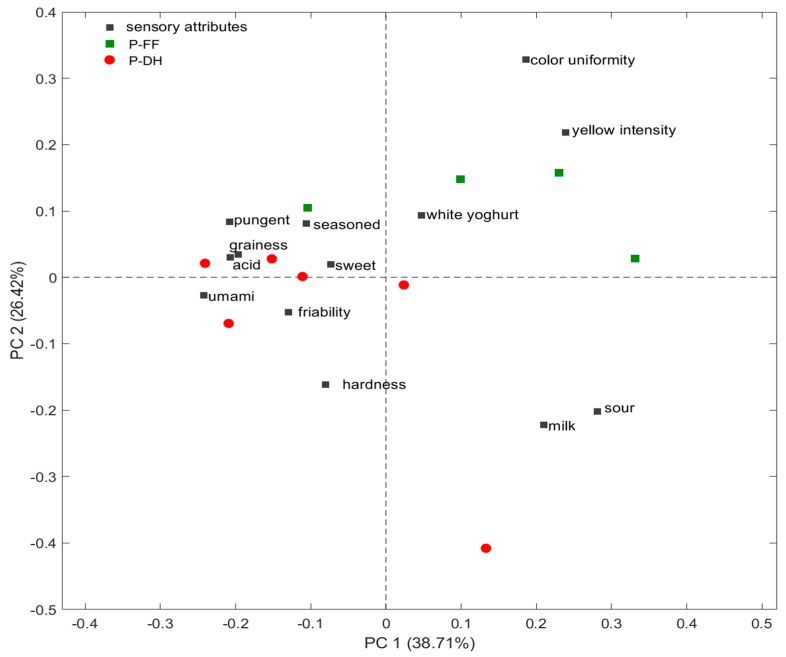
BiPlot of the intensities of sensory attributes and samples of PR PDO cheese. P-DH: PR samples from cows fed with dry hay; P-FF: PR samples from cows fed with a supplementation of fresh herbage.

Moreover, to investigate the possible discrimination among samples, PCA was performed on medians of the score of the intensities of the descriptors for all 10 samples tasted (Figure 3). The principal components PC1 and PC2 explained 65.1%. PC1 (38.7%) was primarily associated with appearance attributes (color uniformity and yellow intensity) and olfactory components like milk and white yogurt. PC2 (26.4%) was related to taste and texture attributes, like umami, sweet, graininess, friability and hardness. Thus, the PCA allowed for a discrimination between P-DH and P-FF samples, despite the moderate explained variance; this could also have been affected by the limited number of samples related to the need to find samples obtained from milk produced by cows fed with fresh grass and cows whose diet consists solely of dry forage.

P-FF samples were associated with the attributes in the first quadrant, such as color uniformity, color intensity, and white yogurt, while most of the P-DH group samples were characterized by descriptors such as umami, acid, and sweet.

### 3.2. Image Analysis

**Figure 4:**
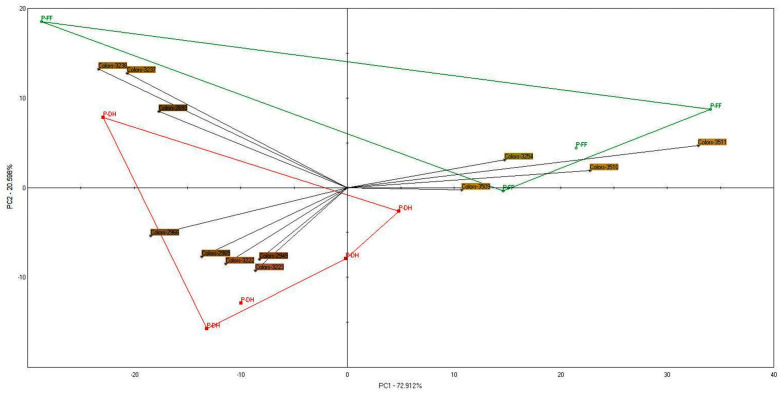
BiPlot (score + loadings) of the colors of the samples of PR PDO. P-DH: PR samples from cows fed with dry hay; P-FF: PR samples from cows fed with a supplementation of fresh herbage.

To discriminate the samples in terms of colors, data collected by “electronic eye” were elaborated using PCA (Figure 4). The different directions and locations of vectors (PCA loadings) show which variables in colors were involved in appearance variation among the samples. In this case, the PR samples were discriminated: those belonging to the P-FF group were positioned in the first and fourth quadrants and exhibited a greater color intensity. Moreover, by using the software of the visual analyzer, it was possible to group the color spectra into 16-bit categories for each RGB coordinate (red, green, and blue) with 4096 variables for analysis. The proportion of each color in the image based on fixed scale of 4096 colors is shown as a percentage. In particular, “color 3254” (L* = 74.858; a* = −2.006; b* = 42.494), “color 3510” (L* = 76.363; a* = 4.065; b* = 44.713), “color 3511” (L* = 76.545; a* = 5.694; b* = 36.925) were associated with the strongest yellow intensities and characterized the P-FF samples.

### 3.3. Flash GC-FID Analysis of the Volatile Fraction

**Figure 5:**
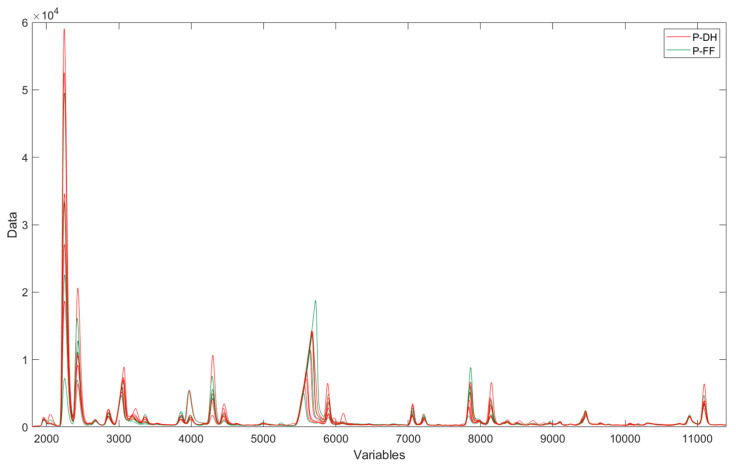
Chromatograms obtained by Flash GC-FID (1st column) of PR PDO cheeses. P-DH: PR samples from cows fed with dry hay; P-FF: PR samples from cows fed with a supplementation of fresh herbage.

**Figure 6:**
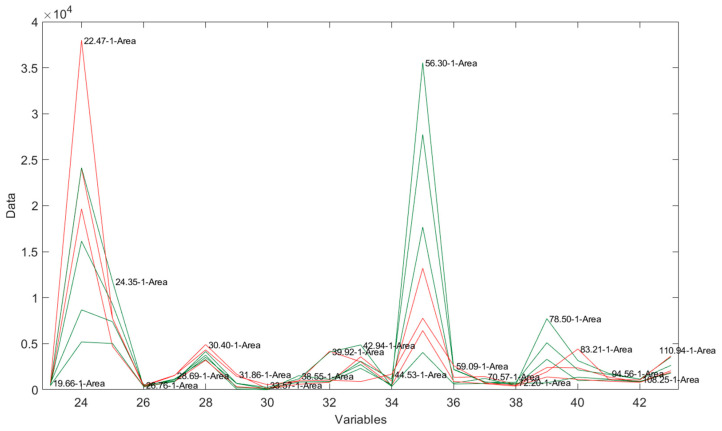
Areas of selected peaks in the chromatograms obtained by Flash GC-FID (1st column) of PR PDO cheese. P-DH (areas in red): PR samples from cows fed with dry hay; P-FF (areas in green): PR samples from cows fed with a supplementation of fresh herbage.

To assess possible differences in the aromatic profile, the samples were analyzed using Flash GC-FID, a very fast chromatographic technique. The chromatograms of the first column of cheese samples are presented in Figure 5. It was observed that the samples displayed chromatographic peaks of varying intensities, but it was not possible to discriminate the samples. The same lack of discrimination based on different diets was observed considering the areas of selected peaks in the chromatograms (Figure 6).

**Figure 7:**
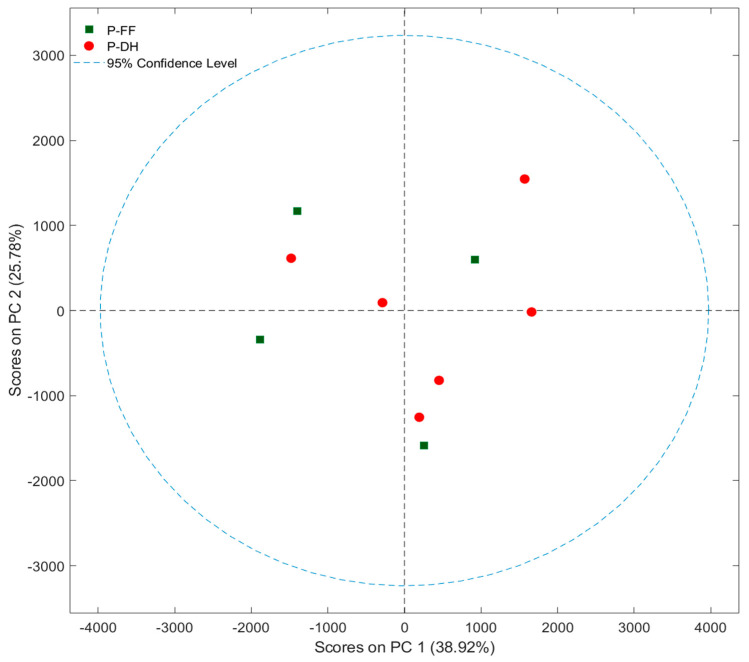
Score plot obtained by PCA of 43 peaks of chromatograms of PR PDO cheese. P-DH: PR samples from cows fed with dry hay; P-FF: PR samples from cows fed with a supplementation of fresh herbage.

Moreover, from the evaluation of the PCA score plot, considering the 43 peaks in the chromatograms for both groups, the samples were not separated according to the different feeding of dairy cows (Figure 7).

## 4. Discussion

Specifically, this study focused on the sensory and instrumental analysis of PR samples, sourced from different dairies, after a 24-month aging period. Of these, six were produced using milk from cows fed dry forage, while four samples were produced with milk from cows fed a diet in which part of the dry hay was replaced with fresh grass. Considering the aroma profile, no significant differences, based on instrumental volatile analysis using Flash GC-FID, emerged between the chromatograms of the two experimental groups (P-DH: PR samples from cows fed with dry hay and P-FF: PR samples from cows fed with supplementation of fresh herbage). Various factors could influence the volatile profile beyond the type of diet provided to the cows. Forage storage, including methods and practices, farming management, cattle breed, season, parity, stage of lactation, and health status of cows, may also contribute to variability [23]. Moreover, PR PDO is produced using milk from cows belonging to different breeds, such as Italian Friesian, Italian Brown, Modena White, and Reggiana Red [3]. In addition, factors like the milk’s microbiological profile, processing factors of cheese with factory conditions, and the aging time could influence the aroma of the final product [7]. In fact, the longer a cheese is aged, the more biochemical reactions will occur, increasing the concentration of volatile compounds within it [5]. On the other hand, the lack of differences in the overall volatile profile of the two experimental cheeses analyzed herein might be due to the fact that PR is a PDO product, and thus standardized to comply with regulations [24,25], given that dairies and production factories must strictly comply with guidelines established by the PR consortium [3]. Therefore, the similarity between the overall aroma profiles reflects this level of general standardization. Moreover, the lack of significant differences may also be attributed to the fact that the instrument has lower sensitivity compared with human senses. Environmental factors such as temperature fluctuations, humidity, and background odors can interfere with sensor function, reducing the precision of the collected data [26].

However, according to the QDA^®^ results, the samples exhibited dairy aroma, but also tastes as umami and acid. Furthermore, the texture of the cheese was more granular and crumblier due to the presence of tyrosine crystals on the surface, a phenomenon favored by an advanced state of proteolysis. In addition, the “sweet” taste strictly associated with samples belonging to the P-DH group could be attributed to the presence of γ-lactones that are linked to sweet notes such as vanilla and caramel [7,27]. A diversification of samples through QDA^®^ could be attributed to the release of volatile compounds in the oral and nasal cavities. This somewhat could be confirmed that an important physiological factor influencing the release of volatile aromatic compounds in the mouth is saliva, given that hydration or dilution of food by saliva affects the distribution of volatile compounds between the food, saliva, and gas phases [28,29]. In fact, the number of bolus fragments increases, which facilitates the release of taste and aroma compounds from the food matrix [30,31]. The release of volatile compounds might be linked to mastication and dilution but could not be detected by a simple headspace analysis of a “static” sample.

Another interesting aspect is that P-FF samples with the same aging period but obtained from cows fed fresh grass supplement showed a more intense yellow color. This difference was perceived both by the panel tasters and through instrumental analysis using image analysis. This result could be attributed to the higher β-carotene content in the milk of PR PDO cheese obtained by dairy cows fed with a supplementation of fresh herbage. Carotenoids could be depleted through photo-oxidation during herbage drying and storage, and the milk’s carotenoid content might be directly related to dietary carotenoid intake [32,33]. Some authors have hypothesized that carotenoids could be a suitable biomarker to differentiate pasture and fresh herbage-produced dairy products from other diets [34,35]. In fact, dried forage could have a lower content of carotenoids due to exposure to ultraviolet rays [36,37,38]. This present work also confirms that color is considered as a distinguishing element of the type of diet of dairy cows. In fact, the more intense yellow color of Parmigiano Reggiano from cows fed with fresh forage could serve as a preference driver in markets where consumers favor cheeses with more pronounced yellow hues. This aspect may prove relevant in determining which product is best suited for export to different countries of the world. Furthermore, understanding these sensory attributes is crucial for effectively disseminating information about the product, particularly among consumers who may be less familiar with it [8].

## 5. Conclusions

The characterization of the PR PDO samples analyzed herein highlighted differences in terms of sensory profiles. Although rapid instrumental analysis of the aroma profile revealed a strong similarity among the samples, indicating a high degree of standardization in line with the strict PDO regulations, it is interesting that olfactory differences could be observed in the sensory evaluation (“white yogurt and milk” attributes in P-FF group) than in the specific variations of volatile compounds. This may be due to the fact that headspace analysis does not yield results that are fully comparable with those obtained during mastication and dilution. Moreover, the attribute “yellow intensity” emerged as the most distinctive parameter among samples, as identified through sensory evaluation, using QDA^®^, and image analysis using the electronic eye. Beyond its influence on consumer preferences, this attribute also could be a valuable marker for differentiating PR PDO cheese derived from milk from cows whose diet included fresh grass supplementation. Therefore, such characterization could be useful for dairy industries, with the aim of developing a product that meets specific preferences and contributing to the authenticity of this high-value food. Moreover, it would be necessary to collect a larger number of samples to conduct more reliable analyses; however, this is challenging, as it is difficult to find milk samples from milk produced by cows fed solely with dry forage or supplemented with fresh herbage. For future studies, it would be interesting to further investigate the aromatic profile, particularly through olfactory sensory perception using GC-MS combined with olfactometry method, to evaluate the possible correlations between sensory and instrumental data. It would be interesting to expand this study by applying an in vitro mastication process, examining the release of aromas during the chewing process, and identifying the volatile compounds that characterize the sensory profile of cheeses with target analysis.

## Figures and Tables

**Table 1:** Consensus list of attributes describing appearance, aroma, flavor, taste, and mouthfeel sensations of samples. Attributes were used to construct the sensory profile sheet for the PR PDO cheeses analyzed herein.

Attribute	Definition	Standard	Anchor Point (Intensity on a 100 mm Unstructured Scale)
*Appearance*			
Color intensity	Visual characteristic evaluated from the freshly cut surface that measures the intensity of the specific color shade	Pantones of color chosen based on the type of cheese	50/100
Color uniformity	Visual characteristic evaluated on a freshly cut surface that measures the degree of color unevenness and the presence of areas with different colors compared with the predominant color	Images of cheese sections identifying different degrees of color uniformity.	50/100
*Aroma and flavor*			
Milk	Olfactory characteristic belonging to the “fresh milk” family, reminiscent of milk, butter, curd, and fresh cheeses	Pasteurized milk	100/100
White yogurt	Olfactory characteristic belonging to the “acidified milk” family, reminiscent of sour whey, cream, and yogurt	Low-fat natural yogurt	100/100
Seasoned	Olfactory characteristic reminiscent of aged cheese	Grated hard cheese aged for minimum 24 months	70/100
*Taste/mouthfeel*			
Sweet	Gustatory sensation caused by sugars such as sucrose	-	-
Salty	Gustatory sensation caused by inorganic salts such as sodium chloride	-	-
Sour	Gustatory sensation caused by acidic substances such as lactic acid	-	-
Umami	Gustatory sensation caused by substances such as monosodium glutamate or certain nucleotides	-	-
Pungent	Trigeminal gustatory sensation corresponding to a tingling (fine needles), perceived in the oral cavity and throat during chewing, caused by substances such as pepper	-	-
*Texture*			
Hardness	Mechanical characteristic that measures the resistance of the sample to light pressure exerted by the molars before deformation or breaking	-	-
Friability	Characteristic that measures the ease with which a sample breaks into numerous fragments from the beginning of mastication (after a predetermined number of chews)	-	-
Graininess	Characteristic that measures the ease of perception of particles (granules) formed in the sample before it is reduced to a bolus (after a defined number of chews)	-	-

## Data Availability

The original contributions presented in this study are included in the article. Further inquiries can be directed to the corresponding author.
